# Supplementary material for: Probing the activated complex of the F + NH3 reaction via a dipole-bound state
Source: Nat Commun. 2024 May 8;15:3858. doi: 10.1038/s41467-024-48202-7 (PMC11079065; doi:10.1038/s41467-024-48202-7)
Supplement: Supplementary file 1 — Supplementary Information [file 41467_2024_48202_MOESM1_ESM.pdf]

Supplementary Information

Probing the activated complex of the $F + NH_3$ reaction via a dipole-bound state

Rui Zhang[1], Shuaiting Yan[1], Hongwei Song[2*], Hua Guo[3], and Chuangang Ning[1*]

1 Department of Physics, State Key Laboratory of Low Dimensional Quantum Physics, Frontier Science Center for Quantum Information, Tsinghua University, Beijing 100084, China

2 State Key Laboratory of Magnetic Resonance Spectroscopy and Imaging, Innovation Academy for Precision Measurement Science and Technology, Chinese Academy of Sciences, Wuhan 430071, China

3 Department of Chemistry and Chemical Biology, Center for Computational Chemistry, University of New Mexico, Albuquerque, New Mexico 87131, USA

Corresponding Authors

* hwsong@wipm.ac.cn, ningcg@tsinghua.edu.cn

Contents

Supplementary Experimental Data **S3**
Supplementary Computational Methods **S3**
Quantum dynamics calculations S3
DBS calculations S5
Supplementary Notes **S5**
Assignments of peaks *0a-0j* S5
Assignments of peaks *1a-1f* S6
Supplementary Tables and Figures **S7**
Figure 1. Jacobi coordinates S7
Figure 2a. 2D cuts of the *0a-0j* wavefunctions S8
Figure 2b. 2D cuts of the *1a-1f* wavefunctions S9
Figure 3. Photoelectron yields as a function of photon energy S10
Table 1. Electron binding energies, widths, shifts and assignments of all peaks S11
Table 2. Numerical parameters used in the calculations S12
Table 3. Harmonic frequencies for the NH_2-HF well on the neutral PES S13
Table 4. Extra 6*s*6*p* diffusive functions for DBS calculations S14
Supplementary References **S15**

Supplementary Experimental Data

The electron binding energies, widths and shifts of all resonance peaks are provided in Supplementary Table 1.

Supplementary Computational Methods

1. Quantum dynamics calculations

The quantum dynamics calculations were carried out within a seven-dimensional model. Supplementary Fig. 1 shows the nine-dimensional diatom-triatom (AB-CDE) Jacobi coordinates employed in this work. Under the approximation of fixing the two non-reactive NH bond lengths, the full-dimensional Hamiltonian is reduced to a seven-dimensional one, as shown in the main text.

The parity ε adapted wavefunction of the system was expanded in terms of body-fixed (BF) rovibrational basis functions:

$$\psi^{J_{tot}M\varepsilon}(\vec{R},\vec{r}_1,\vec{r}_{2e},\vec{r}_{3e}) = \sum_{n,v_1,j,K} F^{J_{tot}M\varepsilon}_{nv_1jK}\, u_n(R)\, \varphi_{v_1}(r_1)\Phi^{J_{tot}M\varepsilon}_{jK}(\hat{R},\hat{r}_1,\hat{r}_{2e},\hat{r}_{3e}), \quad \text{(S1)}$$

where $u_n(R)$ represents the translational sine basis function along the translational coordinate R, $\varphi_{v_1}(r_1)$ denotes the vibrational basis function along r_1, which is defined as the eigenfunction of the one-dimensional (1D) reference Hamiltonian, $\hat{h} = -\frac{1}{2\mu_{r_1}}\frac{\partial^2}{\partial r_1^2} + V^{ref}(r_1)$ with $V^{ref}(r_1)$ as the 1D reference potential. J_{tot} is the total angular momentum of the system and set to zero in the simulation. The composite index j denotes the rotational bases $(j_1, l_2, j_3, j_{23}, J)$, in which j_3 represents the rotational angular momentum of DE and l_2 the orbital angular momentum of atom C with respect to DE, j_{23} is coupled by j_3 and l_2, j_1 is rotational angular momentum of AB, and J is coupled by j_1 and j_{23}. $\Phi^{J_{tot}M\varepsilon}_{jK}$ in Eq. (S1) is the parity-adapted eigenfunction of the total angular momentum operator ($\hat{J}_{tot} = 0$), and is expressed as

$$\Phi^{J_{tot}M\varepsilon}_{jK} = (1+\delta_{K0})^{-1/2}\sqrt{\frac{2J_{tot}+1}{8\pi}}\,[D^{*J_{tot}}_{K,M} Y^{JK}_{j_1j_{23}l_2j_3} + \varepsilon(-1)^{j_1+l_2+j_3+J+J_{tot}} D^{*J_{tot}}_{-K,M} Y^{J-K}_{j_1j_{23}l_2j_3}], \quad \text{(S2)}$$

where $D^{J_{tot}}_{K,M}$ denotes the Wigner rotation matrix. M and K the projections of the total angular momentum on the space-fixed and BF z axes, respectively. The BF z axis was defined to be along with the coordinate R. $Y^{JK}_{j_1j_{23}l_2j_3}$ is the eigenfunction of $\hat{J}$, which is defined as

$$Y^{JK}_{j_1j_{23}l_2j_3} = \sum_\omega \langle j_1\omega j_{23}K-\omega|JK\rangle\, y_{j_1\omega}(\hat{r}_1)Y_{j_{23}l_2j_3K-\omega}(\hat{r}_{2e},\hat{r}_{3e}), \quad \text{(S3)}$$

and

$$Y_{j_{23}l_2j_3K-\omega}(\hat{r}_{2e},\hat{r}_{3e}) = \sum_m \bar{D}^{j_{23}}_{K-\omega m}(\hat{r}_{2e})\sqrt{\frac{2l_2+1}{2j_{23}+1}}\langle j_3ml_20|j_{23}m\rangle y_{j_3m}(\hat{r}_{3e}), \quad \text{(S4)}$$

where ω is the projection of j_1 on the BF z axis R and m is the projection of j_3 on r_2. y_{jm} denotes the spherical harmonics. The angular bases must satisfy the condition $\varepsilon(-1)^{j_1+l_2+j_3+J+J_{tot}} = 0$ for $K = 0$ in Eq. (S2).

The initial wave packet $|\chi_i\rangle$ was obtained by diagonalizing the seven-dimensional Hamiltonian on the anion PES, in which the parallel ARPACK software package[1] was utilized. The anion ground rovibrational state was then placed vertically on the neural PES and propagated in the Chebyshev order domain[2]:

$$|\psi_{k+1}\rangle = D(2\hat{H}_{scaled}|\psi_k\rangle - D|\psi_{k-1}\rangle), \quad k \geq 1, \quad \text{(S5)}$$

where $|\psi_1\rangle = D\hat{H}_{scaled}|\psi_0\rangle$ and $|\psi_0\rangle = |\chi_i\rangle$. To evaluate the action of the Hamiltonian onto the wave packet, the Hamiltonian needs to be normalized to the range [-1, 1] as $\hat{H}_{scaled} = (\hat{H} - H^+)/H^-$ to avoid the divergence of the Chebyshev propagator. Here the spectral medium and half-width of the discretized Hamiltonian were calculated by $H^{\pm} = (H_{max} \pm H_{min})/2$ with H_{min} and H_{max} being the spectral extrema[3]. The action of the Hamiltonian matrix onto the wave packet was efficiently evaluated by transforming the wave packet between the finite basis representation and discrete variable representation[4]. D is a damping function to enforce outgoing boundary conditions, which was defined as $D(x) = e^{-\alpha\left(\frac{x-x_a}{x_{max}-x_a}\right)^n}$, where x_a is the starting point of the damping function and x_{max} is the maximum of the corresponding grid.

The energy spectrum was calculated from the Chebyshev autocorrelation function $C_k = \langle\psi_0|\psi_k\rangle$:

$$S(E) = \frac{1}{\pi H^- sin\theta}\sum_{k=0}(2 - \delta_{k,0})\,cos(k\theta)C_k\,, \quad \text{(S6)}$$

where $\delta_{k,0}$ denotes the Kronecker delta and the Chebyshev angle is given by $\theta = arccos(E - H^+)/H^-$. In addition, the raw spectrum was broadened by including a window function, exp $(-\beta^2k^2)$, in Eq. (S6) to mimic the finite experimental resolution.

The wavefunction at the energy E was computed by

$$|\psi(E)\rangle = \frac{1}{2\pi H^- sin\theta}\sum_k\left(2 - \delta_{k,0}\right)cos(k\theta)|\psi_k\rangle. \quad \text{(S7)}$$

The numerical parameters used in the calculations are provided in Supplementary Table 2. All the parameters were carefully checked to give converged results.

2. DBS calculations

The dipole moment of FNH_3 was calculated with the density functional theory (DFT) using the double hybrid density functional B2PLYP combined with Grimme's D3BJ dispersion[5, 6]. The dipole moment of FNH_3 in its ground state was calculated using at the equilibrium geometry, while the vibrationally averaged geometry was used to calculate the dipole moment for $v_{HF} = 1$. The basis sets aug-cc-pVTZ[7, 8] were used for all the atoms. The calculations mentioned above were performed with the Gaussian program[9]. The calculated dipole moment was 4.8 D for FNH_3 in its ground state, and 5.0 D for $v_{HF} = 1$. The properties for the DBS of FNH_3^- were calculated using the equation-of-motion coupled-cluster method for electron attachment with single and double excitations (EOM-EA-CCSD)[10-12] via the Q-Chem program[13]. The basis sets aug-cc-pVTZ extended with the extra diffuse functions 6*s*6*p* in an even-tempered manner were used to describe the diffusive nature of DBS. Our calculations showed that FNH_3^- has a σ-type DBS. The calculated electron binding energy of the DBS is 327 cm^{-1} for $v_{HF} = 0$, and 415 cm^{-1} for $v_{HF} = 1$. The extra 6*s*6*p* functions are listed in Supplementary Table 4.

Supplementary Notes

Most peaks in the photoelectron spectrum can be unambiguously assigned by plotting the two-dimensional (2D) cuts of the corresponding wavefunctions along different coordinates. The harmonic frequencies of the PC complex ($NH_2 \cdot HF$) minima listed in Supplementary Table 3 helped us to assign the observed peaks. As discussed below, there are mainly three vibrational modes of $HF \cdot NH_2$ (v_1 (v_{HF}), v_6, v_8) involved in the photodetachment over the interested energy region, the three quantum numbers denote excitations in the H-F stretching mode (v_1 (v_{HF}) = 3674 cm^{-1}), the pseudo-rotational mode of HF coupled with the out-of-plane wagging of NH_2 (v_6 = 829 cm^{-1}) and the H_2N-HF stretching mode (v_8 = 251 cm^{-1}). The onsets of the vibrational bands of v_{HF} = 0, 1 and 2 were theoretically estimated by the sum of the corresponding excitation energy of the 1D Hamiltonian along the coordinate r_1 and the ground-state energy of the neutral $HF \cdot NH_2$ complex, which are 20079, 23456, and 26527 cm^{-1}, respectively.

1. Peaks *0a-0j*

Figure 4 in the main text shows the 2D cuts of the wave functions corresponding to peaks *0a-0j* and *1a-1f* along the coordinates R and r_1. Since there exist no node for peaks *0a-0j* along the H-F bond (r_1), the H-F stretching mode is unexcited and these peaks are assigned with $v_{HF} = 0$. For peaks *0a-0c*, there exist zero, one and two nodes along R, respectively, indicating different excitations of the H_2N-HF stretching mode. Therefore, peaks *0a-0c* were assigned with v_8 = 0, 1, and 2, respectively. Similarly, there exist zero, one

and two nodes along R for peaks *0d-0f*, respectively, and one, two, three and four nodes for peaks *0g-0j*, respectively. Peaks *0d-0f* were thus assigned with $v_8 = 0$, 1, and 2, respectively and peaks *0g-0j* with $v_8 = 1$, 2, 3, and 4, respectively. Supplementary Figure 2a shows 2D cuts of the wavefunctions along different angular coordinates. For peaks *0a-0c*, there is no node along the angular coordinates and these peaks were assigned with $v_6 = 0$. For peaks *0d-0f*, there exists one node along the coordinate φ_2, indicating the excitation of modes related to the out-of-plane motion. According to the normal mode analysis, only the modes, v_6 and v_7, are associated with the out-of-plane motion. The two modes are both formed by the coupling of the pseudo-rotational mode of HF with the out-of-plane wagging of NH_2, with the corresponding harmonic frequencies being 829 and 318 cm^{-1}, respectively. Considering the energy gap between peaks *0a* and *0d* is 565 cm^{-1} (and similarly from peak *0b* to *0e* and from *0c* and *0f*), peaks *0d-0f* were assigned with $v_6 = 1$. The assignments of peaks *0g-0j* are somewhat tentative. It appears that there exist two nodes along the coordinate θ_2 except peak *0i* and one peak along the coordinate φ_2. These peaks were assigned with $v_6 = 2$ in combination with the energy gap. Supplementary Table 1 lists the assignments of all these peaks based on the nodal structures of the wavefunctions and energies.

2. Peaks *1a-1f*

As shown in Fig.4 in the main text, there exists one mode along the coordinate r_1 for peaks *1a-1f*. These peaks were hence assigned with $v_{HF} = 1$. The wave functions of peaks *1a* and *1b* display zero and one node along R, respectively, and thus the two peaks were assigned with $v_8 = 0$ and 1, respectively. Similarly, peaks *1c-1e* were assigned with $v_8 = 0$, 1 and 2 and peak *1f* with $v_8 = 0$. The two cuts of the wavefunctions along the different angular coordinates are plotted in Supplementary Figure 2b. Clearly, there is no node along the angular coordinates for peaks *1a* and *1b* and the two peaks were assigned with $v_6 = 0$. There exists one node along the coordinate φ_2 for peaks *1c-1e* and the three peaks were assigned with $v_6 = 1$ in combination with the energy gap. In turn, peak *1f* was assigned with $v_6 = 2$.

Supplementary Tables and Figures

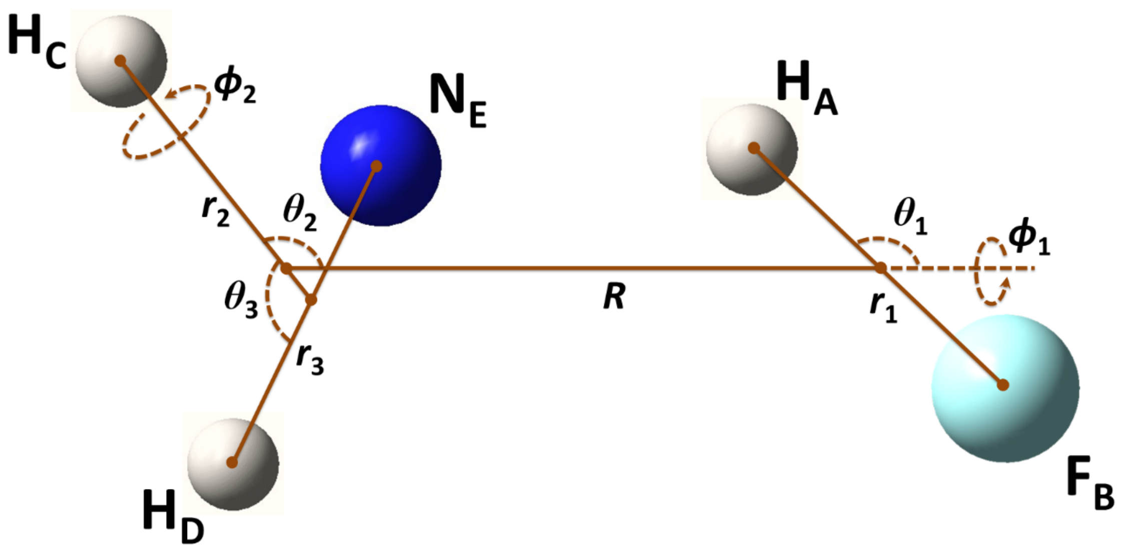

Supplementary Figure 1. 2+3 Jacobi coordinates of the diatom-triatom (AB-CDE) system used in the quantum dynamical calculations.

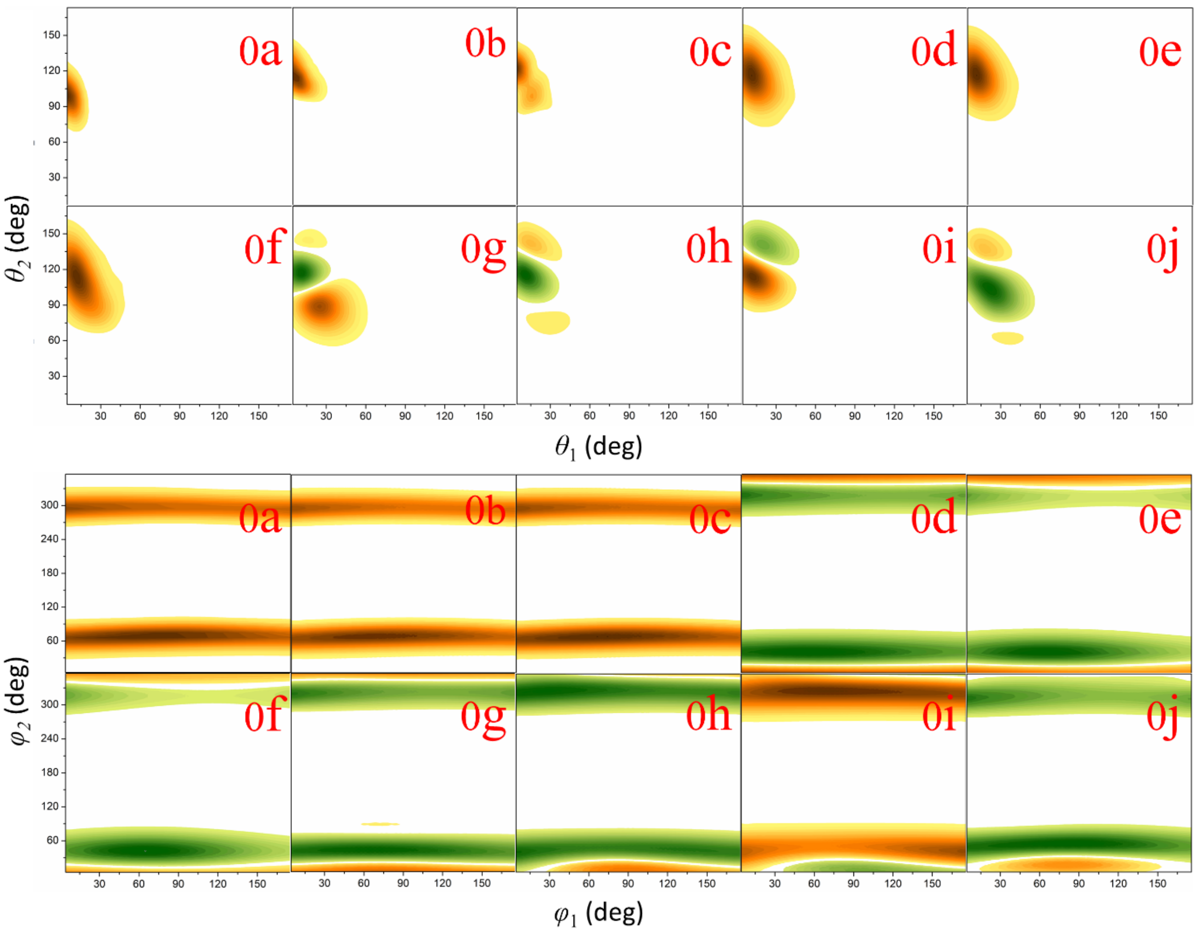

Supplementary Figure 2a. 2D cuts of the wavefunctions of peaks *0a-0j* along different angular coordinates, as defined in Supplementary Figure 1.

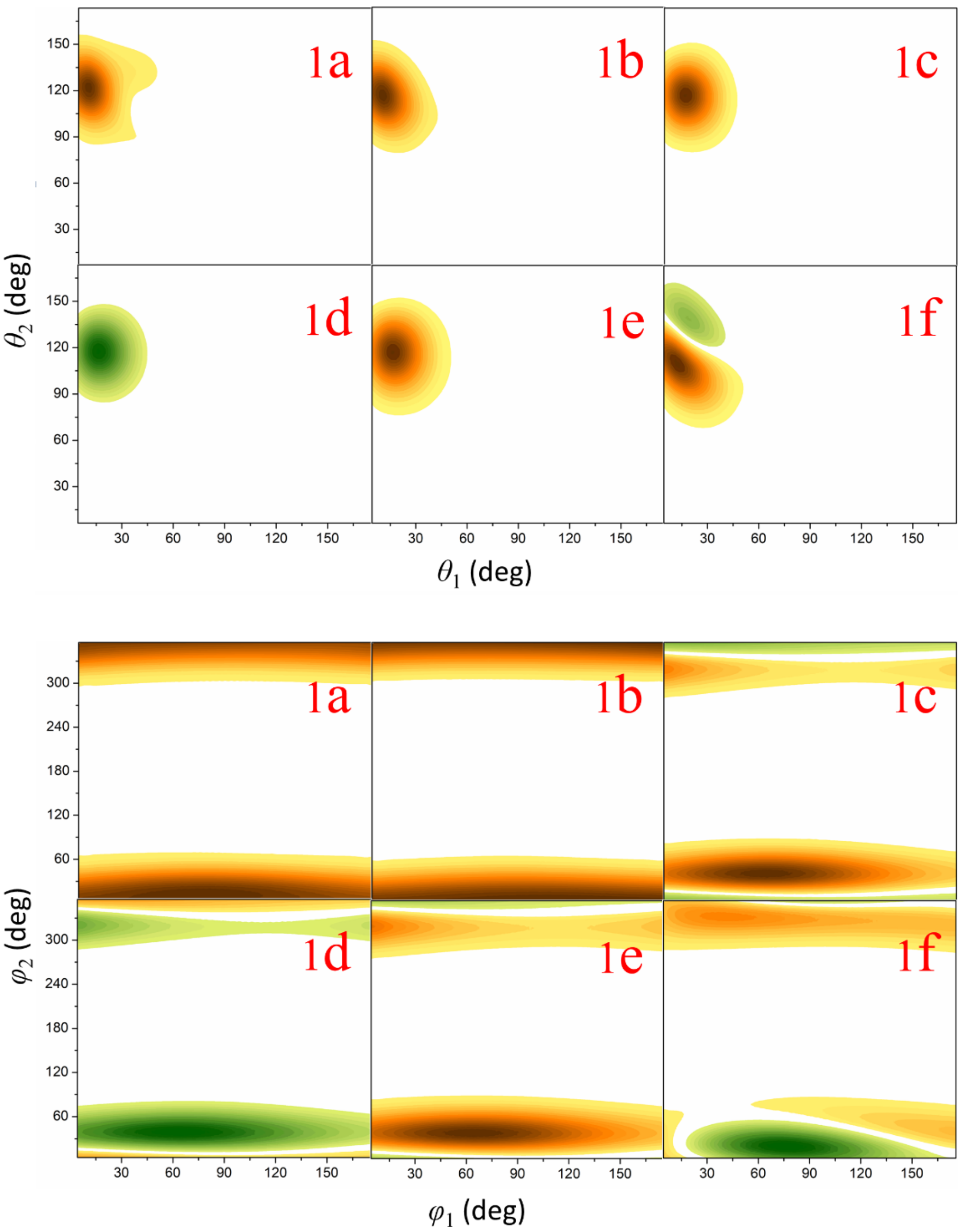

Supplementary Figure 2b. 2D cuts of the wavefunctions of peaks *1a*-*1f* along different angular coordinates, as defined in Supplementary Figure 1.

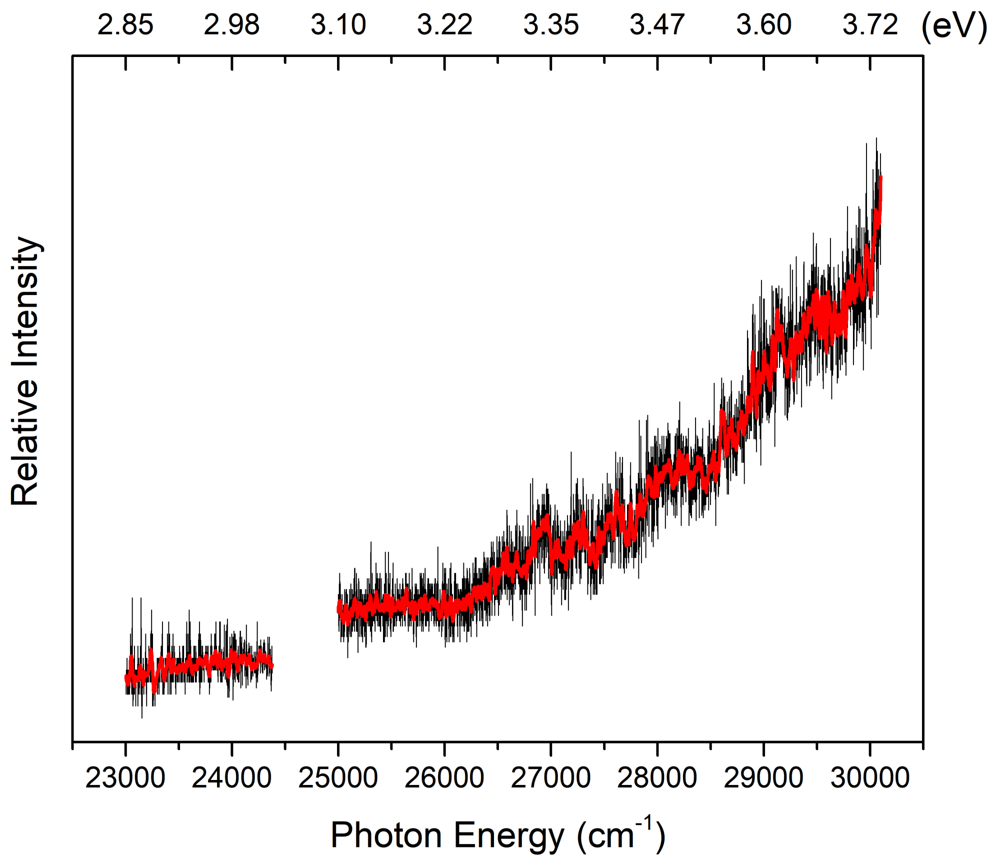

Supplementary Figure 3. Photoelectron yields as a function of photon energy. The red curve is the smoothened one. The broken region at ~24600 cm^{-1} is due to the dead band of our laser system.

Supplementary Table 1. Electron binding energies, widths and shifts of all resonance peaks observed in the present work. The peaks are labeled the same way as in the figures. Three vibrational modes, namely the HF stretching mode (v_1 (v_{HF}) = 3674 cm^{-1}), the NH_2 out-of-plane wag mode (v_6 = 829 cm^{-1}), and H_2N-HF stretching mode (v_8 = 251 cm^{-1}), of the HF-NH_2 complex (PC) are used to assign all observed features.

Peaks	Expt eBE (cm^{-1})	Expt FWHM (cm^{-1})	Expt Shift (cm^{-1})	Theo Shift (cm^{-1})	Assignment (ν_1, ν_6, ν_8)
0a	20220	89	0	0	(0,0,0)
0b	20505	89	285	221	(0,0,1)
0c	20724	108	504	426	(0,0,2)
0d	20840	82	620	574	(0,1,0)
0e	21074	77	854	783	(0,1,1)
0f	21291	171	1071	973	(0,1,2)
0g	21475	73	1255	1208	(0,2,1)
0h	21648	58	1428	1388	(0,2,2)
0i	21821	70	1601	1558	(0,2,3)
0j	21969	83	1749	1709	(0,2,4)
1a	23713	207	3493	3470	(1,0,0)
1b	24098	221	3878	3719	(1,0,1)
1c	24360	139	4140	4088	(1,1,0)
1d	24607	180	4387	4321	(1,1,1)
1e	24841	138	4621	4536	(1,1,2)
1f	25076	249	4856	4829	(1,2,1)
2a	26877	125	6657	6581	(2,0,0)
2b	27165	145	6945	6871	(2,0,1)
2c	27474	152	7254	7213	(2,1,0)
2d	27766	202	7546	7484	(2,1,1)
2e	28065	181	7845	7838	(2,2,0)
2f	28359	94	8139	8098	(2,2,1)
2g	28639	98	8419	8432	(2,3,0)
2h	28908	112	8688	8660	(2,3,1)
2i	29166	60	8946	8934	(2,4,0)
2j	29422	106	9202	9133	(2,4,1)

Supplementary Table 2. Numerical parameters used in the Chebyshev wave packet calculations (atomic units are used unless stated otherwise).

Grid/basis range and size:	$R \in [3.5, 11.5], N_R = 73$<br>$r_1 \in [0.8, 6.0], N_{r_1} = 32$<br>$j_{1max} = 30, l_{2max} = 20\ , j_{3max} = 22, j_{23max} = 46, J_{max} = 24$
Damping potential:	$R_a = 8.0, R_{max} = 11.5, \alpha_R = 0.5, n_R = 2.0$<br>$r_{2a} = 4.8, r_{2max} = 6.0, \alpha_{r_2} = 0.025, n_{r_2} = 1.5$
Window function	$\beta = 0.00075$
Propagation steps:	5000

Supplementary Table 3. Harmonic frequencies for the NH_2-HF complex (PC) on the neutral PES. ν_1 (ν_{HF}): HF stretching; ν_2: NH_2 asymmetric stretching; ν_3: NH_2 symmetric stretching; ν_4: NH_2 scissoring; ν_5: pseudo rotation of HF coupled with in-plane rocking of NH_2; ν_6: pseudo rotation of HF coupled with out-of-plane wagging of NH_2; ν_7: out-of-plane wagging of NH_2 coupled with pseudo rotation of HF; ν_8: H_2N-HF stretch; ν_9: in-plane rocking of NH_2 coupled with pseudo rotation of HF.

PC	Frequency (cm^{-1})
ν_1	3674
ν_2	3522
ν_3	3425
ν_4	1555
ν_5	844
ν_6	829
ν_7	318
ν_8	251
ν_9	197

Supplementary Table 4. The extra 6*s*6*p* diffusive functions added to the standard aug-cc-pVTZ basis sets in an even-tempered manner for N, H, and F atoms for the DBS calculations. The data listed here are in the Gaussian format. The exponents α_k were generated using the equation $\alpha_{k+1} = \alpha_0 \varepsilon^k$, k = 1-6. α_0 was obtained from the same type functions with the smallest exponent in the aug-cc-pVTZ basis sets, and ε was the ratio of the smallest two exponents.

		N	H	F
6s	1	S 1 1.00<br>0.01856608 1.00	S 1 1.00<br>0.00621300 1.00	S 1 1.00<br>0.027579403 1.00
	2	S 1 1.00<br>0.00598436 1.00	S 1 1.00<br>0.00152812 1.00	S 1 1.00<br>0.008305563 1.00
	3	S 1 1.00<br>0.00192892 1.00	S 1 1.00<br>0.00037586 1.00	S 1 1.00<br>0.002501228 1.00
	4	S 1 1.00<br>0.000621748 1.00	S 1 1.00<br>9.24453D-05 1.00	S 1 1.00<br>0.000753247 1.00
	5	S 1 1.00<br>0.000200407 1.00	S 1 1.00<br>2.27378E-05 1.00	S 1 1.00<br>0.000226841 1.00
	6	S 1 1.00<br>6.45967D-05 1.00	S 1 1.00<br>5.59256E-06 1.00	S 1 1.00<br>6.83134D-05 1.00
6p	1	P 1 1.00<br>0.01397571 1.00	P 1 1.00<br>0.0268144 1.00	P 1 1.00<br>0.020278563 1.00
	2	P 1 1.00<br>0.003978014 1.00	P 1 1.00<br>0.00704915 1.00	P 1 1.00<br>0.005586471 1.00
	3	P 1 1.00<br>0.001132293 1.00	P 1 1.00<br>0.00185312 1.00	P 1 1.00<br>0.001538997 1.00
	4	P 1 1.00<br>0.000322293 1.00	P 1 1.00<br>0.00048716 1.00	P 1 1.00<br>0.000423973 1.00
	5	P 1 1.00<br>9.17368D-05 1.00	P 1 1.00<br>0.00012806 1.00	P 1 1.00<br>0.000116799 1.00
	6	P 1 1.00<br>2.61117D-05 1.00	P 1 1.00<br>3.36675D-05 1.00	P 1 1.00<br>3.21765D-05 1.00

Supplementary References:

1. Maschhoff, K.J. & Sorensen, D.C. in Applied Parallel Computing Industrial Computation and Optimization 478-486 (Springer, Berlin; 1996).
2. Chen, R. & Guo, H. Evolution of quantum system in order domain of chebyshev operator. *J. Chem. Phys.* **105**, 3569-3578 (1996).
3. Tal-Ezer, H. & Kosloff, R. An accurate and efficient scheme for propagating the time dependent Schrödinger equation. *J. Chem. Phys.* **81**, 3967-3971 (1984).
4. Light, J.C. & Carrington Jr., T. in Adv. Chem. Phys. 263-310 (2000).
5. Grimme, S. Semiempirical hybrid density functional with perturbative second-order correlation. *J. Chem. Phys.* **124**, 034108 (2006).
6. Grimme, S., Ehrlich, S. & Goerigk, L. Effect of the damping function in dispersion corrected density functional theory. *J. Comp. Chem.* **32**, 1456-1465 (2011).
7. Prascher, B.P., Woon, D.E., Peterson, K.A., Dunning, T.H. & Wilson, A.K. Gaussian basis sets for use in correlated molecular calculations. VII. Valence, core-valence, and scalar relativistic basis sets for Li, Be, Na, and Mg. *Theor. Chem. Acc.* **128**, 69-82 (2011).
8. Woon, D.E. & Dunning, T.H., Jr. Gaussian basis sets for use in correlated molecular calculations. III. The atoms aluminum through argon. *J. Chem. Phys.* **98**, 1358-1371 (1993).
9. Frisch, M. et al. Gaussian 09, Revision D. 01, Gaussian, Inc., Wallingford CT. *See also: URL: http://www.gaussian.com* (2009).
10. Krylov, A.I. Equation-of-motion coupled-cluster methods for open-shell and electronically excited species: the hitchhiker's guide to fock space. *Annu. Rev. Phys. Chem.* **59**, 433-462 (2008).
11. Sneskov, K. & Christiansen, O. Excited state coupled cluster methods. *WIREs Comput. Mol. Sci.* **2**, 566-584 (2012).
12. Nooijen, M. & Bartlett, R.J. Equation of motion coupled cluster method for electron attachment. *J. Chem. Phys.* **102**, 3629-3647 (1995).
13. Shao, Y. et al. Advances in molecular quantum chemistry contained in the Q-Chem 4 program package. *Mol. Phys.* **113**, 184-215 (2015).
